# Cannabinoids Alleviate the LPS-Induced Cytokine Storm via Attenuating NLRP3 Inflammasome Signaling and TYK2-Mediated STAT3 Signaling Pathways In Vitro

**DOI:** 10.3390/cells11091391

**Published:** 2022-04-20

**Authors:** Santosh V. Suryavanshi, Mariia Zaiachuk, Nazar Pryimak, Igor Kovalchuk, Olga Kovalchuk

**Affiliations:** Department of Biological Sciences, University of Lethbridge, Lethbridge, AB T1K 3M4, Canada; mariia.zaiachuk@uleth.ca (M.Z.); nazar.pryimak@uleth.ca (N.P.); igor.kovalchuk@uleth.ca (I.K.)

**Keywords:** delta-9-tetrahydrocannabinol, cannabidiol, cannabinoids, NLRP3 inflammasome, STAT3, TYK2, cytokine storm, interleukins, TNF-α, macrophages, primary lung bronchial epithelial cells

## Abstract

Cannabinoids, mainly cannabidiol (CBD) and Δ^9^-tetrahydrocannabinol (THC), are the most studied group of compounds obtained from *Cannabis sativa* because of their several pharmaceutical properties. Current evidence suggests a crucial role of cannabinoids as potent anti-inflammatory agents for the treatment of chronic inflammatory diseases; however, the mechanisms remain largely unclear. Cytokine storm, a dysregulated severe inflammatory response by our immune system, is involved in the pathogenesis of numerous chronic inflammatory disorders, including coronavirus disease 2019 (COVID-19), which results in the accumulation of pro-inflammatory cytokines. Therefore, we hypothesized that CBD and THC reduce the levels of pro-inflammatory cytokines by inhibiting key inflammatory signaling pathways. The nucleotide-binding and oligomerization domain (NOD)-like receptor family pyrin domain-containing 3 (NLRP3) inflammasome signaling has been implicated in a variety of chronic inflammatory diseases, which results in the release of pyroptotic cytokines, interleukin-1β (IL-1β) and IL-18. Likewise, the activation of the signal transducer and activator of transcription-3 (STAT3) causes increased expression of pro-inflammatory cytokines. We studied the effects of CBD and THC on lipopolysaccharide (LPS)-induced inflammatory response in human THP-1 macrophages and primary human bronchial epithelial cells (HBECs). Our results revealed that CBD and, for the first time, THC significantly inhibited NLRP3 inflammasome activation following LPS + ATP stimulation, leading to a reduction in the levels of IL-1β in THP-1 macrophages and HBECs. CBD attenuated the phosphorylation of nuclear factor-κB (NF-κB), and both cannabinoids inhibited the generation of oxidative stress post-LPS. Our multiplex ELISA data revealed that CBD and THC significantly diminished the levels of IL-6, IL-8, and tumor necrosis factor-α (TNF-α) after LPS treatment in THP-1 macrophages and HBECs. In addition, the phosphorylation of STAT3 was significantly downregulated by CBD and THC in THP-1 macrophages and HBECs, which was in turn attributed to the reduced phosphorylation of tyrosine kinase-2 (TYK2) by CBD and THC after LPS stimulation in these cells. Overall, CBD and THC were found to be effective in alleviating the LPS-induced cytokine storm in human macrophages and primary HBECs, at least via modulation of NLRP3 inflammasome and STAT3 signaling pathways. The encouraging results from this study warrant further investigation of these cannabinoids in vivo.

## 1. Introduction

According to the Public Health Agency of Canada, millions of Canadians suffer from chronic diseases, also called noncommunicable diseases (NCDs), which account for the highest causes of death (88% of all deaths) in Canada [1,2]. NCDs include a variety of chronic diseases, such as cancer, arthritis, diabetes, cardiovascular disorders, chronic respiratory diseases, and mood disorders. Additionally, 44% of Canadian adults (age of 20 years and older) have at least one of the above-mentioned chronic conditions, including osteoporosis and dementia [3]. Moreover, the treatment cost of chronic diseases in Canada is approximately $190 billion annually, which is approximately 58% of the country’s annual healthcare spending [4].

Almost all chronic diseases or NCDs can be classified as chronic inflammatory diseases, given that chronic inflammation is a vital contributor to their pathophysiology. Acute inflammation is the body’s defense response to harmful external pathogens and is a crucial part of our innate immunity. However, unhindered acute inflammation, persistent acute inflammatory signals, and sterile inflammation result in chronic inflammation [5]. Various chronic diseases, such as diabetes, autoimmune disorders, obesity, and inflammatory bowel disorders, are linked to chronic inflammation and elevated circulating levels of pro-inflammatory cytokines in the body despite their short half-lives. This constant pathological rise in cytokines, termed cytokine storm or cytokine release syndrome, can result in systemic effects causing organ damage if it is not controlled [6]. In the milieu of the current coronavirus disease 2019 (COVID-19) pandemic, people with pre-existing chronic inflammatory conditions are susceptible to acute respiratory distress syndrome (ARDS), which is characterized by elevated levels of cytokines after severe acute respiratory syndrome coronavirus 2 (SARS-CoV-2) infection [7]. The cytokine release in chronic inflammation is governed by several complex signaling pathways with considerable overlap, which explains the substantial side effects of approved cytokine inhibitors [8]. Hence, targeted anti-inflammatory drugs that can curb cytokine storms are needed for the treatment of NCDs along with ARDS in COVID-19.

Signaling pathways governed by the nucleotide-binding and oligomerization domain-like receptor family, pyrin domain-containing 3 (NLRP3) inflammasome, and signal transducer and activator of transcription-3 (STAT-3) are implicated in different chronic inflammatory diseases along with induction of cytokine release in COVID-19 [5,9,10,11,12,13,14]. The NLRP3 protein is one of the important pattern recognition receptors that detect conserved microbial structures called pathogen-associated molecular patterns (PAMPs) and host-derived sterile damage (or danger)-associated molecular patterns (DAMPs) to mount an innate immune response. Once the first signal from PAMPs and/or DAMPs is detected, nontranscriptional or transcriptional priming via nuclear factor-κB (NF-κB) causes increased expression of NLRP3. Next, multiple secondary signals, such as cathepsin released from lysosomal rupture or in response to mitochondrial oxidative stress, among others [15], lead to the formation and activation of NLRP3 inflammasome. Once initiated, canonical NLRP3 inflammasome activation results in the formation of active caspase-1, which in turn leads to the cleavage of pro-interleukin-1β (pro-IL-1β) and pro-IL-18 and the release of mature cytokines. The release of mature IL-1β and IL-18 causes cell death by pyroptosis [5]. Although physiological NLRP3 inflammasome activation is important in innate immunity, its pathophysiological activation is implicated in many chronic inflammatory diseases, including cancers and cardiovascular diseases, and in triggering cytokine storms in COVID-19 [5,10]. Similarly, STAT3 is a transcription factor that induces the expression of numerous pro-inflammatory cytokines, including IL-17, IL-23, and IL-8, among others, once activated [12]. Most importantly, IL-6 is a vital STAT-3 activator during inflammation, and the IL-6-STAT3 axis has been identified as a critical target for treating COVID-19-induced cytokine storm [11,13]. In non-immune and immune cells, cytokines such as IL-6, IL-1β, and tumor necrosis factor-alpha (TNF-α) and the transcription factors NF-κB and STAT3 play an important role in the pathogenesis of chronic inflammation. Under the settings of chronic inflammation, elevated cytokines, especially IL-6, activate the Janus kinase (JAK)/STAT pathway, mainly STAT3. JAK is a family of tyrosine kinases, and an increased expression of its member, tyrosine kinase-2 (TYK2), has been recently implicated in life-threatening COVID-19 in a genome-wide analysis of 2636 patients [16]. Once JAKs, such as TYK2, phosphorylate cytosolic STAT3, it results in nuclear translocation of the latter, leading to increased cytokine release. Moreover, NF-κB interacts synergistically with STAT3, causing the hyperactivation of NF-κB, followed by an increased production of inflammatory cytokines, such as IL-6. As more IL-6 is generated, it causes sequential activation of STAT3 and NF-κB, triggering a positive feedback loop of NF-κB activation via the IL-6-JAK-STAT3 pathway. This feedback loop is termed the IL-6 amplifier, which is a key player in autoimmune disorders, oncogenesis, and COVID-19-mediated ARDS and multiorgan failure [11,13,14]. Targeting NLRP3 inflammasome and STAT3 signaling would be crucial to curb cytokine storm.

*Cannabis sativa* (*C. sativa*) is an essential plant originating from Asia and has been utilized extensively in ancient medicine. Cannabinoids are the most important pharmacologically active ingredients extracted from *C. sativa*, in which cannabidiol (CBD) and Δ^9^-tetrahydrocannabinol (THC) are the most studied. Attention and the published literature regarding several applications of cannabinoids, including their potent anti-inflammatory effects, have been growing in the last decade. Our laboratory recently published that certain high CBD cannabis extracts significantly reduced the gene expression of pro-inflammatory cytokines in 3D tissue models of inflammation [17]. The available literature endorses the immunosuppressive role of CBD via different mechanisms, including the induction of apoptosis, activation of immune cells, and reduction of pro-inflammatory cytokines [18,19]. Current data also indicate that CBD inhibits NLRP3 inflammasome activation via the inhibition of NF-κB priming and may modulate the JAK/STAT pathway by limiting the levels of pro-inflammatory cytokines [5,20,21]. Although several previous studies suggest the pro-inflammatory potential of THC [22], growing new evidence indicates the potent anti-inflammatory effects of THC via the induction of immunosuppressive regulatory T cells and suppression of cytokine storm, among other mechanisms [23,24]. However, despite the crucial role of NLRP3 and STAT-3 signaling in the development of cytokine storms and documented evidence of CBD and THC in lowering elevated levels of cytokines, no study has clearly determined whether CBD and THC curb cytokine storms via the inhibition of these pathways in vitro and/or in vivo. Hence, in this manuscript, we attempted to study the molecular mechanisms behind curbing the lipopolysaccharide (LPS)-induced cytokine release by CBD and THC in human THP-1 macrophages and primary human lung bronchial epithelial cells (HBECs).

## 2. Materials and Methods

### 2.1. Chemicals and Reagents

CBD (C-045), Δ^9^-THC (T4764), LPS from *Escherichia coli* O111:B4 (L4391), and adenosine 5′-triphosphate (ATP) disodium salt hydrate (A6419) were obtained from Sigma Aldrich, Oakville, ON, Canada. Phorbol-12-myristate-13-acetate (PMA) was purchased from Enzo (BML-PE160-0005), Farmingdale, NY, USA. CBD and THC were provided as 1 mg/mL stock solutions in methanol and stored at −20 °C. Both cannabinoids were chosen to be used at a final concentration of 5 µM based on our optimization and previously published results [25,26]. LPS was prepared by dissolving 1 mg powder in 1 mL of sterile phosphate buffer saline (PBS) to obtain 1 mg/mL solution as per the manufacturer’s instructions. PMA was dissolved in dimethyl sulfoxide (DMSO), cell culture reagent (CAS 67-68-5) [sc-358801, Santa Cruz Biotechnology (SCBT), Dallas, TX, USA] and further diluted to appropriate concentrations using sterile PBS. Trypan blue solution, 0.4% (15250061) was acquired from ThermoFisher Scientific (Life Technologies Inc., Burlington, ON, Canada).

### 2.2. Cell Culture and Treatments

The THP-1 (ATCC TIB-202) cells were purchased from American Type Culture Collection (Rockville, MD, USA) and cultured as a suspension in Roswell Park Memorial Institute Medium (RPMI-1640) (Cat# 350-000-CL, Wisent Inc., Saint-Jean-Baptiste, QC, Canada) supplemented with 10% heat-inactivated premium-grade fetal bovine serum (FBS) (97068-085, VWR International, Edmonton, AB, Canada) and 1% penicillin-streptomycin antibiotic (LS15140122, Gibco, Life Technologies Inc., Burlington, ON, Canada). Primary normal human bronchial epithelial cells (HBECs) (NHBE-CRY) were purchased from MatTek Life Sciences (MatTek, Ashland, MA, USA) and thawed in the basal medium (NHBE-BM, MatTek) supplemented with growth supplement (NHBE-GS, MatTek) and hydrocortisone (NHBE-HCS, MatTek). The cells were isolated from a healthy Caucasian 59-year-old female by MatTek and shipped frozen as is. The optimum growing conditions were set for 37 °C for at 5% CO_2_–95% O_2_ in the humidified incubator (ThermoFisher Scientific). THP-1 cells were used only from passages 4–7 for all experiments, and regarding HBECs, all experiments were performed from the stock vial of cells without re-freezing and not including hydrocortisone in the media. The THP-1 cells were terminally differentiated into macrophages by adding PMA at a final concentration of 50 ng/mL for 48 h. After two days, cells were rested for 24 h in fresh PMA-free complete RPMI-1640 media, as suggested elsewhere [27]. LPS (0.5 µg/mL) was then added for 3 h, followed by ATP (5 mM) for 1 h in fresh medium wherever indicated. HBECs were cultured and subcultured as per the manufacturer’s instructions using Trypsin-EDTA (0.05%, Gibco), and soybean trypsin inhibitor (SBTI; ATCC 30-2104). LPS (0.5 µg/mL) was used for 8 h followed by ATP (5 mM) for 1 h in a fresh medium wherever indicated. CBD and THC were added 30 min before adding LPS.

### 2.3. Immunoblotting

After respective treatments, cells were harvested using cell lysis buffer (Cell Signaling Technology (CST), 9803, Danvers, MA, USA) by adding 1 mM phenylmethylsulfonyl fluoride (PMSF) immediately before use followed by brief sonication. Cell debris was then cleared by centrifugation at 10,000 g for 2 min [28], and total cell lysates were then stored at −80 °C freezer. Protein concentrations were determined using Bradford assay [29], and about 45–50 μg of proteins were loaded per sample for sodium dodecyl sulfate-polyacrylamide gel electrophoresis (SDS-PAGE) using 10% resolving and 4% stacking gels. The resolved proteins were then transferred to polyvinylidene difluoride (PVDF) membranes (Amersham Hybond^®^ P, GE Healthcare) for 2 h on ice. The PVDF membranes were subsequently blocked with 5% nonfat dry milk prepared in 1X Tris-buffered saline, 0.1% Tween^®^ 20 Detergent (TBST) for at least 2 h at room temperature (RT). Next, membranes were incubated with appropriate primary antibodies at specified concentrations overnight at 4 °C, followed by three washes with 0.1% TBST and incubation with respective horseradish peroxidase (HRP)-conjugated secondary antibodies (1:2500, SCBT) for at least 2 h at RT. Immunoreactivity was visualized using SuperSignal™ West Atto Ultimate Sensitivity Substrate (A38555, ThermoFisher Scientific) for low abundant proteins and UltraScence Pico Ultra Western Substrate (CCH345-B, FroggaBio, Concord, ON, Canada) for all other proteins. To probe for phosphorylated and total proteins, mild stripping buffer (15 g glycine, 1 g SDS, 10 mL Tween 20, pH 2.2 in 1 L distilled water, Abcam) was used per the manufacturer’s instructions. National Institutes of Health (NIH) ImageJ software was utilized for densitometric analyses, and three to four independent technical replications were used for statistical analyses [28]. The primary antibodies were used as mentioned below (Table 1).

### 2.4. Immunoblotting for Mature IL-1β

To detect a mature form of IL-1β in the cell culture supernatants, we optimized the methanol-chloroform precipitation protocol, as explained here [30]. In brief, 100 μL of cell culture supernatant was mixed with a 2:1 mixture of methanol:chloroform to precipitate protein pellets and reconstituted with 50 μL 1X SDS sample buffer. Next, samples were incubated at 95 °C for 5 min, and SDS-PAGE was performed as explained above.

### 2.5. Gene Expression

Total RNA was extracted using 5 mL of TRIzol^®^ Reagent (15596018, Invitrogen, Life Technologies Inc., Burlington, ON, Canada) per 100 mm of the cell-culture plate as per the manufacturer’s instructions. The RNA purity and yield were determined by Nanodrop (ThermoFisher Scientific). The cDNA was synthesized using iScript™ Select cDNA Synthesis Kit (1708897, BioRad Laboratories, Saint-Laurent, QC, Canada) by utilizing 500 ng of total RNA sample according to the manufacturer’s protocols. The generated cDNA was then employed to perform quantitative real-time polymerase chain reaction (qRT-PCR) using SsoFast™ EvaGreen^®^ Supermix (1725200, BioRad Laboratories) in duplicates using the CFX96 Touch™ Real-Time PCR Detection System (Bio-Rad). Data were analyzed using the 2^−ΔΔCT^ method and GAPDH was used to normalize gene expression. The following primers used for our experiments were ordered from Eurofins (Ottawa, ON, Canada) (Table 2).

### 2.6. Multiplex Enzyme-Linked Immunosorbent Assay (ELISA)

Cell culture supernatant (media) samples from all experiments were collected after cannabinoids and LPS treatment and centrifuged at 3000× *g* at 4 °C to remove debris. Next, the samples were aliquoted and stored in a −80 °C freezer. The frozen aliquots were shipped to Eve Technologies Corp. (Calgary, AB, Canada) on dry ice, and all samples were analyzed for the levels of cytokines immediately upon thawing. Thirteen different cytokines were measured using Luminex™ 200 system (Luminex, Austin, TX, USA) by Eve Technologies Corp. by utilizing Human Focused 13-Plex Discovery Assay^®^ (Millipore Sigma, Burlington, MA, USA) according to the manufacturer’s protocol. Out of 13, six cytokines displayed measurable levels in our samples. To quantify IL-1β, we used Human IL-1 beta/IL-1F2 Quantikine ELISA Kit (DLB50, R&D systems, Toronto, ON, Canada); as for this experiment, cell culture supernatant samples were obtained after LPS + ATP treatment. The assay was performed as per the manufacturer’s instructions, and the absorbance was measured at 450 nm with a correction at 540 nm. A standard curve was plotted using standard IL-1β supplied with the kit; Assay Blaster software (ADI-28-00020, Enzo) was used to calculate the concentration of all samples. Each experiment included four biological replicates, and an average of at least two technical replicates was considered for one biological replicate. The THP-1 cell supernatant samples treated with LPS + ATP were diluted 4 times due very high absorbance of released IL-1β.

### 2.7. Reactive Oxygen Species (ROS) Generation Assay

The ROS generation following LPS stimulation and the effects of cannabinoids on the same was assessed by ROS indicator 2′,7′-dichlorodihydrofluorescein diacetate (H2DCFDA) using Cellular ROS Assay Kit (ab113851, Abcam, Toronto, ON, Canada). Briefly, 0.5–1 × 10^6^ cells/mL were plated in a 12-well plate for THP-1 cells and in a 96-well plate for HBECs. In the case of HBECs, cells were treated as per indicated times, with diluted DCFDA (20 μM) added 45 min before the completion of experiments. Cells were incubated at 37 °C in the dark, and the fluorescence was measured by a microplate reader with Ex/Em = 485/535 nm. In the case of THP-1 cells, cells were first incubated with 20 μM DCFDA for 45 min at 37 °C in the dark, followed by washings and treatment. Cells were then harvested in 95% DMSO, and fluorescence was immediately measured in a 96-well plate. All readings were subtracted from the background reading of untreated cells to calculate relative fluorescence intensity.

### 2.8. Trypan Blue Cell Viability Assay

The trypan blue dye exclusion test is based on the principle that viable cell membranes are intact, thus excluding the dye leaving clear cytoplasm, whereas nonviable cells take up the dye, staining the cytoplasm blue in color [31]. In brief, when the cells were ready, they were washed with PBS after removing media, and trypsin-EDTA was added. Fresh RPMI-1640 media was added for THP-1 cells, and SBTI was added for HBECs to neutralize trypsin, followed by centrifugation at 1200 rpm for 4 min. The cell pellet was resuspended in fresh media after removing the supernatant, and then an aliquot of suspended cells was mixed with trypan blue in a 1:1 ratio. The viable and nonviable cells were counted on a LUNA-I automated cell counter (Logos Biosystems, Annandale, VA, USA), and % cell viability values were calculated. For each group, three independent experiments were performed in duplicate.

### 2.9. Statistics

The data collected were analyzed by one-way analysis of variance (ANOVA) with post hoc Tukey’s multiple-comparison tests (GraphPad Prism 6). Data were reported as means ± standard deviation (SD). For significance, a *p*-value less than 0.05 was considered statistically significant in all cases.

## 3. Results

### 3.1. CBD and THC Inhibit the Expression of NLRP3 Inflammasome Proteins in THP-1 Cells and HBECs to Downregulate the Expression of Mature IL-1β

**Figure 1:**
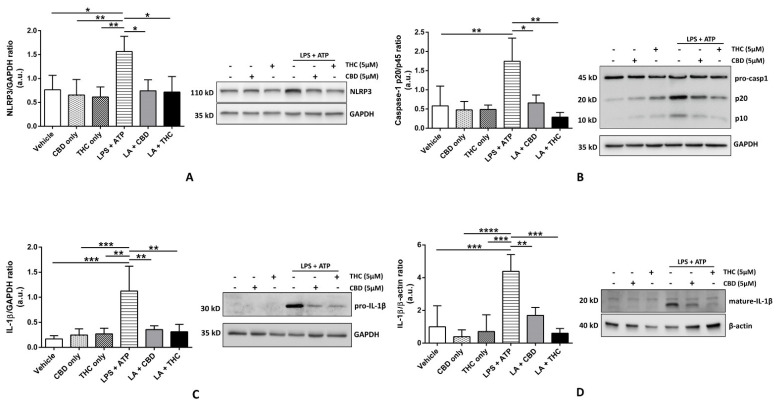
Effect of cannabinoids on the expression of NLRP3 inflammasome proteins in THP-1 macrophages. (**A**): CBD and THC decreased the expression of NLRP3 after LPS + ATP stimulation in THP-1 macrophages: Representative Western blots of NLRP3 and GAPDH bands and the quantification of the same using one-way ANOVA with Tukey’s multiple-comparison test. Four independent experiments (*n* = 3–4) of THP-1 cell lysates obtained after pre-treatment with CBD or THC for 30 min and LPS stimulation for 3 h followed by ATP for 1 h were analyzed, and data are expressed as means ± SD; * *p* < 0.05, ** *p* < 0.01. (Abbreviation: LA: LPS + ATP). (**B**): CBD and THC decreased the ratio of the expressions of caspase-1 p20/p45 after LPS + ATP stimulation in THP-1 macrophages: Representative Western blots of caspase-1 p45 (pro-caspase1), p20, and GAPDH bands and the quantification of caspase-1 p20/p45 ratio using one-way ANOVA with Tukey’s multiple-comparison test. Four independent experiments (*n* = 4) of THP-1 cell lysates obtained after pre-treatment with CBD or THC for 30 min and LPS stimulation for 3 h followed by ATP for 1 h were analyzed, and data are expressed as means ± SD; * *p* < 0.05, ** *p* < 0.01. (Abbreviation: LA: LPS + ATP). (**C**): CBD and THC decreased the expression of pro-IL-1β after LPS + ATP stimulation in THP-1 macrophages: Representative Western blots of pro-IL-1β and GAPDH bands and the quantification of the same using one-way ANOVA with Tukey’s multiple-comparison test. Four independent experiments (*n* = 4) of THP-1 cell lysates obtained after pre-treatment with CBD or THC for 30 min and LPS stimulation for 3 h followed by ATP for 1 h were analyzed, and data are expressed as means ± SD; ** *p* < 0.01, *** *p* < 0.001. (Abbreviation: LA: LPS + ATP). (**D**): CBD and THC decreased the expression of mature IL-1β after LPS + ATP stimulation in cell supernatants of THP-1 macrophages: Representative Western blots of mature-IL-1β and β-actin bands and the quantification of the same using one-way ANOVA with Tukey’s multiple-comparison test. Four independent experiments (*n* = 4) of THP-1 cell supernatants obtained after pre-treatment with CBD or THC for 30 min and LPS stimulation for 3 h followed by ATP for 1 h were analyzed, and data are expressed as means ± SD; ** *p* < 0.01, *** *p* < 0.001, **** *p* < 0.0001. (Abbreviation: LA: LPS + ATP).

**Figure 2:**
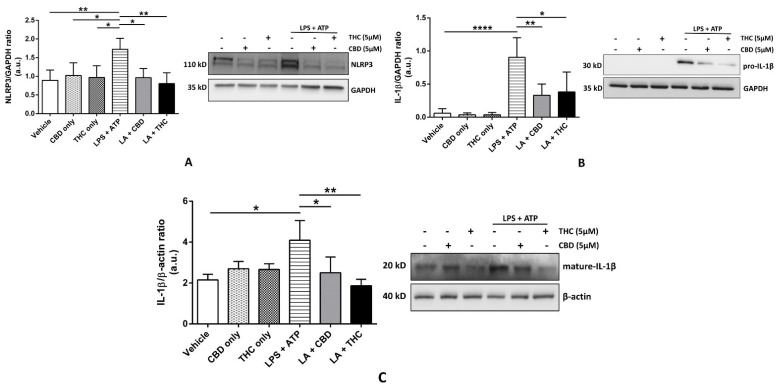
Effect of cannabinoids on the expression of NLRP3 inflammasome proteins in primary human bronchial epithelial cells. (**A**): CBD and THC decreased the expression of NLRP3 after LPS + ATP stimulation in primary HBEC: Representative Western blots of NLRP3 and GAPDH bands and the quantification of the same using one-way ANOVA with Tukey’s multiple-comparison test. Four independent experiments (*n* = 4) of primary human bronchial epithelial cell lysates obtained after pre-treatment with CBD or THC for 30 min and LPS stimulation for 8 h followed by ATP for 1 h were analyzed, and data are expressed as means ± SD; * *p* < 0.05, ** *p* < 0.01. (Abbreviations: HBECs: Human bronchial epithelial cells; LA: LPS + ATP). (**B**): CBD and THC decreased the expression of pro-IL-1β after LPS + ATP stimulation in primary HBECs: Representative Western blots of pro-IL-1β and GAPDH bands and the quantification of the same using one-way ANOVA with Tukey’s multiple-comparison test. Four independent experiments (*n* = 4) of primary human bronchial epithelial cell lysates obtained after pre-treatment with CBD or THC for 30 min and LPS stimulation for 8 h followed by ATP for 1 h were analyzed, and data are expressed as means ± SD; * *p* < 0.05, ** *p* < 0.01, **** *p* < 0.0001. (Abbreviations: HBECs: Human bronchial epithelial cells; LA: LPS + ATP). (**C**): CBD and THC decreased the expression of mature IL-1β after LPS + ATP stimulation in cell supernatants of primary HBECs: Representative Western blots of mature-IL-1β and β-actin bands and the quantification of the same using one-way ANOVA with Tukey’s multiple-comparison test. Four independent experiments (*n* = 4) of primary human bronchial epithelial cell supernatants obtained after pre-treatment with CBD or THC for 30 min and LPS stimulation for 8 h followed by ATP for 1 h were analyzed, and data are expressed as means ± SD; * *p* < 0.05, ** *p* < 0.01. (Abbreviations: HBECs: Human bronchial epithelial cells; LA: LPS + ATP).

To study the effects of CBD and THC on the expression of NLRP3 inflammasome proteins, we pre-treated THP-1 cells and HBECs with CBD and THC, followed by LPS + ATP to induce inflammasome activation. In THP-1 cells, we discovered that CBD and, for the first time, THC, significantly blocked the expression of NLRP3, pro-caspase-1, and pro-IL-1β after LPS + ATP treatment (Figure 1A–C). In particular, the expression of pro-IL-1β was markedly downregulated by CBD and THC (*p* < 0.01); hence, we sought to determine the expression of mature IL-1β in cell culture supernatants. Our data revealed that higher expression of the mature form of IL-1β after LPS + ATP treatment was also markedly inhibited by CBD and THC (Figure 1D). We observed somewhat analogous results in HBECs. The expression of NLRP3 and pro-IL-1β was significantly downregulated by CBD and THC after LPS + ATP stimulation in HBECs (Figure 2A,B). However, we did not find a significant difference in the expression of pro-caspase-1 across all experimental groups (data not shown). When we performed immunoblotting for mature IL-1β in cell culture supernatants, the increased expression of the mature form of IL-1β after LPS + ATP stimulation was significantly abrogated by CBD and THC in HBECs (Figure 2C). Unfortunately, we could not detect cleaved caspase-1 expression in our cell lysates and cell culture supernatants. Nevertheless, our immunoblotting data uncovered that CBD and THC could abolish NLRP3 inflammasome activation by decreasing the expression of NLRP3 inflammasome-associated proteins.

### 3.2. CBD, but Not THC, Diminishes the Phosphorylation of NF-κB p-65 Subunit at Ser-536, Thereby Reducing the Expression of Inflammasome Proteins in THP-1 Cells and HBECs

**Figure 3:**
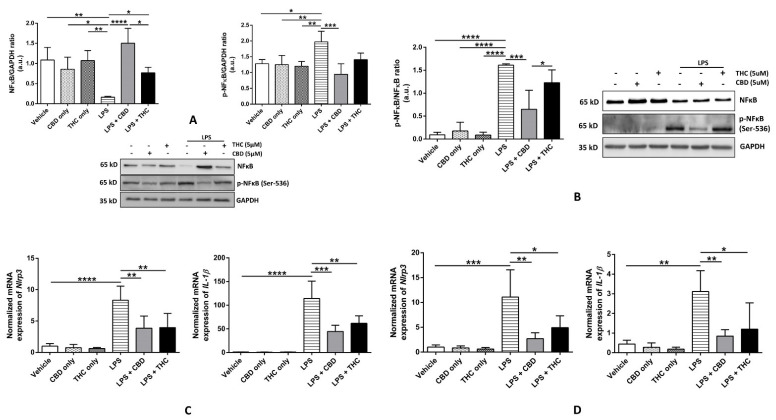
Effect of cannabinoids on the NFκB-mediated gene expression in THP-1 macrophages and primary human bronchial epithelial cells. (**A**): CBD, not THC, decreased the expression of phospho-NFκB p65 (Ser-536) after LPS stimulation in THP-1 macrophages: Representative Western blots of NFκB, p-NFκB, and GAPDH bands and the quantification of the same using one-way ANOVA with Tukey’s multiple-comparison test. Four independent experiments (*n* = 4) of THP-1 cell lysates obtained after pre-treatment with CBD or THC for 30 min and LPS stimulation for 3 h were analyzed, and data are expressed as means ± SD; * *p* < 0.05, ** *p* < 0.01, *** *p* < 0.001, **** *p* < 0.0001. (**B**): CBD, not THC, decreased the ratio of the expressions of phospho-NFκB p65 (Ser-536)/NFκB after LPS stimulation in primary HBECs: Representative Western blots of NFκB, *p*-NFκB, and GAPDH bands and the quantification of the same using one-way ANOVA with Tukey’s multiple-comparison test. Four independent experiments (*n* = 4) of primary human bronchial epithelial cell lysates obtained after pre-treatment with CBD or THC for 30 min and LPS stimulation for 8 h were analyzed, and data are expressed as means ± SD; * *p* < 0.05, *** *p* < 0.001, **** *p* < 0.0001. (Abbreviation: HBECs: Human bronchial epithelial cells). (**C**): CBD and THC decreased the gene expression of *Nlrp3* and *Il-1β* after LPS stimulation in THP-1 macrophages: The mRNA expression of *Nlrp3* and *Il-1β* relative to GAPDH normalized to vehicle quantified using one-way ANOVA with Tukey’s multiple-comparison test. Four independent experiments (*n* = 4) using total RNA isolated from THP-1 cells obtained after pre-treatment with CBD or THC for 30 min and LPS stimulation for 3 h were analyzed, and data are expressed as means ± SD; ** *p* < 0.01, *** *p* < 0.001, **** *p* < 0.0001. Each independent experiment was run in duplicates. (**D**): CBD and THC decreased the gene expression of *Nlrp3* and *Il-1β* after LPS stimulation in primary HBECs: The mRNA expression of *Nlrp3* and *Il-1β* relative to GAPDH normalized to vehicle was quantified using one-way ANOVA with Tukey’s multiple-comparison test. Four independent experiments (*n* = 4) using total RNA isolated from primary bronchial epithelial cells obtained after by pre-treatment with CBD or THC for 30 min and LPS stimulation for 8 h were analyzed, and data are expressed as means ± SD; * *p* < 0.05, ** *p* < 0.01, *** *p* < 0.001. Each independent experiment was run in duplicates. (Abbreviation: HBECs: Human bronchial epithelial cells).

The phosphorylation of the transcription factor NF-κB and its subsequent nuclear translocation increased the transcription of several pro-inflammatory target genes. The *pro-IL-1β* and *NLRP3* genes are direct targets of NF-κB, and the activation of NF-κB via upstream stimulation of toll-like receptors by LPS serves as a priming signal of NLRP3 inflammasome activation [32,33]. As NF-κB contains multiple subunits [32], we chose to study the phosphorylation of the p-65 subunit at Ser-536 position (p-NFκB) because of its established role in mediating the priming of NLRP3 inflammasome [34]. We discovered that LPS treatment significantly inhibited the expression of total NF-κB (t-NFκB) and upregulated the expression of p-NFκB in THP-1 cells (Figure 3A). As shown by others [35], our data displayed that only CBD, and not THC, reduced the expression of p-NFκB after LPS treatment and substantially increased the expression of t-NFκB (*p* < 0.001) (Figure 3A). The immunoblotting data from HBECs revealed no significant changes in the expression of t-NFκB; however, the expression of p-NFκB was significantly downregulated by CBD, and not by THC (Figure 3B). Given that NFκB is a transcriptional activator of *Nlrp3* and *Il-1β* genes, we measured the normalized expression of these genes in THP-1 cells and HBECs. We noticed that LPS treatment significantly induced the expression of *Nlrp3* and *Il-1β* as compared with the vehicle, and pre-treatment with CBD and THC significantly blocked their expression as compared with LPS in both THP-1 cells and HBECs (Figure 3C,D).

### 3.3. CBD and THC Markedly Reduce the Increased Levels of IL-1β, IL-6, IL-8, and TNF-α after LPS Stimulation in THP-1 Cells and HBECs, Thus Curbing LPS-Mediated Cytokine Release

**Figure 4:**
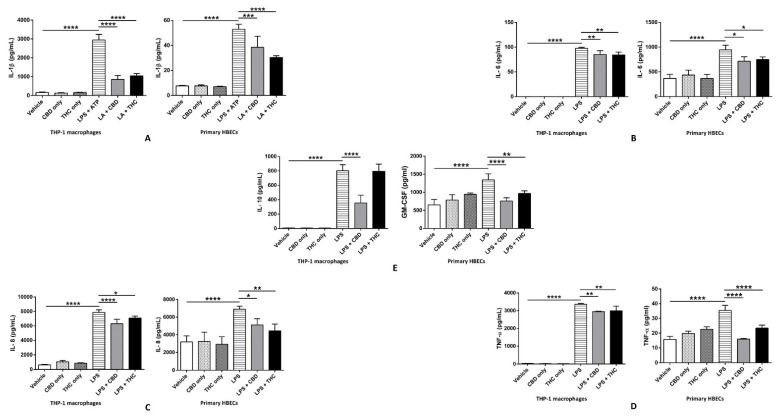
Effect of cannabinoids on the levels of cytokines in THP-1 macrophages and primary human bronchial epithelial cells. (**A**): CBD and THC decreased the levels of IL-1β after LPS + ATP stimulation in THP-1 macrophages and primary HBECs: The levels of released IL-1β in the cell supernatants were measured using ELISA kit (R&D systems) and quantified using one-way ANOVA with Tukey’s multiple-comparison test. Four independent experiments (*n* = 4) from THP-1 and primary human bronchial epithelial cell supernatants obtained after pre-treatment with CBD or THC for 30 min and LPS stimulation (3 h for THP-1 cells and 8 h for primary HBECs) followed by ATP treatment for 1 h were performed, and data are expressed as means ± SD; *** *p* < 0.001, **** *p* < 0.0001. Each independent experiment was run in duplicates. (Abbreviation: LA: LPS + ATP; HBECs: Human bronchial epithelial cells). (**B**): CBD and THC decreased the levels of IL-6 after LPS stimulation in THP-1 macrophages and primary HBECs: The levels of released IL-6 in the cell supernatants were measured using multiplex ELISA and quantified using one-way ANOVA with Tukey’s multiple-comparison test. Four independent experiments (*n* = 4) from THP-1 and primary human bronchial epithelial cell supernatants obtained after pre-treatment with CBD or THC for 30 min and LPS stimulation (3 h for THP-1 cells and 8 h for primary HBECs) were performed, and data are expressed as means ± SD; * *p* < 0.05, ** *p* < 0.01, **** *p* < 0.0001. Each independent experiment was run in duplicates. (Abbreviation: HBECs: Human bronchial epithelial cells). (**C**): CBD and THC decreased the levels of IL-8 after LPS stimulation in THP-1 macrophages and primary HBECs: The levels of released IL-8 in the cell supernatants were measured using multiplex ELISA and quantified using one-way ANOVA with Tukey’s multiple-comparison test. Four independent experiments (*n* = 4) from THP-1 and primary human bronchial epithelial cell supernatants obtained after pre-treatment with CBD or THC for 30 min and LPS stimulation (3 h for THP-1 cells and 8 h for primary HBECs) were performed, and data are expressed as means ± SD; * *p* < 0.05, ** *p* < 0.01, **** *p* < 0.0001. Each independent experiment was run in duplicates. (Abbreviation: HBECs: Human bronchial epithelial cells). (**D**): CBD and THC decreased the levels of TNF-α after LPS stimulation in THP-1 macrophages and primary HBECs: The levels of released TNF-α in the cell supernatants were measured using multiplex ELISA and quantified using one-way ANOVA with Tukey’s multiple-comparison test. Four independent experiments (*n* = 4) from THP-1 and primary human bronchial epithelial cell supernatants obtained after pre-treatment with CBD or THC for 30 min and LPS stimulation (3 h for THP-1 cells and 8 h for primary HBECs) were performed, and data are expressed as means ± SD; ** *p* < 0.01, **** *p* < 0.0001. Each independent experiment was run in duplicates. (Abbreviation: HBECs: Human bronchial epithelial cells). (**E**): CBD decreased the levels of IL-10 after LPS stimulation in THP-1 macrophages whereas CBD and THC decreased the levels of GM-CSF after LPS stimulation in primary HBECs: The levels of released IL-10 and GM-CSF in the cell supernatants were measured using multiplex ELISA and quantified using one-way ANOVA with Tukey’s multiple-comparison test. Four independent experiments (*n* = 4) from THP-1 and primary human bronchial epithelial cell supernatants obtained after pre-treatment with CBD or THC for 30 min and LPS stimulation (3 h for THP-1 cells and 8 h for primary HBECs) were performed and data are expressed as means ± SD; ** *p* < 0.01, **** *p* < 0.0001. Each independent experiment was run in duplicates. (Abbreviation: HBECs: Human bronchial epithelial cells).

The release of IL-1β is a cardinal output of NLRP3 inflammasome activation. We discovered that the levels of IL-1β were significantly upregulated after LPS + ATP stimulation compared with the vehicle, and CBD and THC were able to markedly decrease IL-1β compared with the LPS + ATP group in THP-1 cells and HBECs (Figure 4A). Notably, the rise of IL-1β in immune cells (THP-1 cells) is far greater than that in non-immune cells (HBECs). To compare the effects of CBD and THC on LPS-stimulated cytokine release, we performed a multiplex ELISA. Our data revealed that 0.5 μg/mL of LPS treatment for 3 h markedly upregulated levels of numerous pro-inflammatory cytokines, such as IL-6, IL-8, and TNF-α, in non-immune and immune cells. Interestingly, CBD and THC were able to significantly decrease the levels of IL-6, IL-8, and TNF-α in THP-1 cells and HBECs (Figure 4B–D). Additionally, in HBECs, CBD and THC also diminished the increased levels of granulocyte-macrophage colony-stimulating factor (GM-CSF) after LPS treatment (Figure 4E). However, in THP-1 cells, the elevated levels of IL-10 after LPS stimulation were significantly lowered by CBD but not by THC (Figure 4E). Overall, our data uncovered the potential anti-inflammatory action of CBD and THC via declining the levels of major pro-inflammatory cytokines triggered by LPS stimulation in non-immune and immune cells.

### 3.4. CBD and THC Curb the Cellular ROS Generation Induced by LPS in THP-1 Cells and HBECs

**Figure 5:**
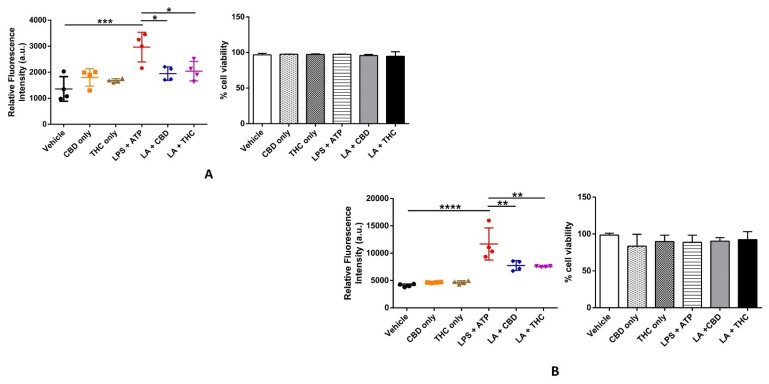
Effect of cannabinoids on ROS generation and cell viability in THP-1 macrophages and primary human bronchial epithelial cells. (**A**): CBD and THC decreased the cellular ROS levels after LPS + ATP stimulation without affecting cell viability in THP-1 macrophages: The ROS levels and % cell viability were measured using cellular ROS assay kit (Abcam) and trypan blue assay, respectively and quantified using one-way ANOVA with Tukey’s multiple-comparison test. Four independent experiments (*n* = 4) for ROS assay and three independent experiments (*n* = 3) for trypan blue assay from THP-1 cells obtained after pre-treatment with CBD or THC for 30 min and LPS stimulation for 3 h followed by ATP 1 h were performed, and data are expressed as means ± SD; * *p* < 0.05, *** *p* < 0.001. Each independent experiment was run in duplicates for both assays. (Abbreviation: LA: LPS + ATP). (**B**): CBD and THC decreased the cellular ROS levels after LPS + ATP stimulation without affecting cell viability in primary HBECs: The ROS levels and % cell viability were measured using cellular ROS assay kit (Abcam) and trypan blue assay, respectively and quantified using one-way ANOVA with Tukey’s multiple-comparison test. Four independent experiments (*n* = 4) for ROS assay and three independent experiments (*n* = 3) for trypan blue assay from primary HBECs obtained after pre-treatment with CBD or THC for 30 min and LPS stimulation for 8 h followed by ATP 1 h were performed, and data are expressed as means ± SD; ** *p* < 0.01, **** *p* < 0.0001. Each independent experiment was run in duplicates for both assays. (Abbreviation: LA: LPS + ATP; HBECs: Human bronchial epithelial cells).

LPS and ATP can both induce ROS-dependent oxidative stress via multiple intracellular mechanisms leading to the activation of caspase-1 and the secretion of IL-1β in macrophages [36,37]. ROS-mediated oxidative stress is one of the well-characterized second signals required for the activation of the NLRP3 inflammasome. Additionally, LPS treatment alone can induce oxidative stress due to cytotoxicity and affects cell viability [38]. Hence, we measured the cellular ROS levels using the fluorogenic cell-permeable dye DCFDA to quantitatively detect hydroxyl, peroxyl, and other ROS levels in live cells and performed a cell viability assay using the dye trypan blue as an indicator of cytotoxicity. We found that, as shown by others [36,37], LPS + ATP treatment significantly increased ROS levels in THP-1 cells as compared with the vehicle, and pre-treatment with CBD and THC substantially lowered the LPS + ATP-induced spike in ROS (Figure 5A). Remarkably, we also discovered that the increased ROS generation induced by LPS + ATP was subdued by CBD and THC in HBECs (Figure 5B). We found no difference in percentage cell viability as measured by trypan blue assay in all experimental groups, indicating that the concentrations of all chemicals used were not cytotoxic to our THP-1 cells and HBECs (Figure 5A,B). These data suggest that CBD and THC noticeably prevent ROS generation initiated by LPS + ATP in our non-immune and immune cells without being cytotoxic to them.

### 3.5. CBD and THC Decrease the Phosphorylation of STAT3 in Part via Reducing the Phosphorylation of TYK2 after LPS Stimulation in HBECs but Not in THP-1 Macrophages

**Figure 6:**
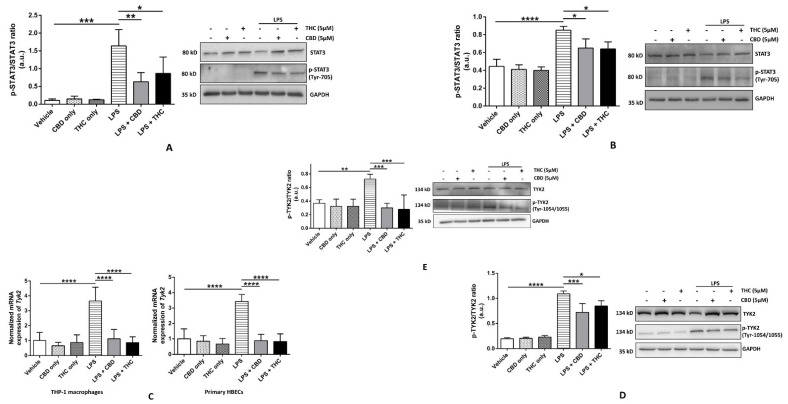
Effect of cannabinoids on TYK-2-mediated phosphorylation of STAT3 in THP-1 macrophages and primary human bronchial epithelial cells. (**A**): CBD and THC decreased the ratio of the expressions of phospho-STAT3 (Tyr-705)/STAT3 after LPS stimulation in THP-1 macrophages: Representative Western blots of p-STAT3, STAT3, and GAPDH bands and the quantification of the ratio p-STAT3/STAT3 using one-way ANOVA with Tukey’s multiple-comparison test. Four independent experiments (*n* = 4) of primary human bronchial epithelial cell lysates obtained after pre-treatment with CBD or THC for 30 min and LPS stimulation for 3 h were analyzed, and data are expressed as means ± SD; * *p* < 0.05, ** *p* < 0.01, *** *p* < 0.001. (**B**): CBD and THC decreased ratio of the expressions of phospho-STAT3 (Tyr-705)/STAT3 after LPS stimulation in primary HBECs: Representative Western blots of p-STAT3, STAT3, and GAPDH bands and the quantification of the ratio p-STAT3/STAT3 using one-way ANOVA with Tukey’s multiple-comparison test. Four independent experiments (*n* = 4) of THP-1 cell lysates obtained after pre-treatment with CBD or THC for 30 min and LPS stimulation for 8 h were analyzed, and data are expressed as means ± SD; * *p* < 0.05, **** *p* < 0.0001. (Abbreviation: HBECs: Human bronchial epithelial cells). (**C**): CBD and THC decreased the gene expression of *Tyk2* after LPS stimulation in THP-1 macrophages and primary HBECs: The mRNA expression of *Tyk2* relative to GAPDH normalized to vehicle was quantified using one-way ANOVA with Tukey’s multiple-comparison test. Four independent experiments (*n* = 4) using total RNA isolated from THP-1 and primary bronchial epithelial cells obtained after by pre-treatment with CBD or THC for 30 min and LPS stimulation (3 h for THP-1 cells and 8 h for primary HBECs) were analyzed, and data are expressed as means ± SD; **** *p* < 0.0001. Each independent experiment was run in duplicates. (Abbreviation: HBECs: Human bronchial epithelial cells). (**D**): CBD and THC decreased the ratio of the expressions of phospho-TYK2 (Tyr1054/1055)/TYK2 after LPS stimulation in THP-1 macrophages: Representative Western blots of p-TYK2, TYK2, and GAPDH bands and the quantification of the ratio p-TYK2/TYK2 using one-way ANOVA with Tukey’s multiple-comparison test. Four independent experiments (*n* = 4) of THP-1 cell lysates obtained after pre-treatment with CBD or THC for 30 min and LPS stimulation for 3 h were analyzed, and data are expressed as means ± SD; * *p* < 0.05, *** *p* < 0.001, **** *p* < 0.0001. (**E**): CBD and THC decreased the ratio of the expressions of phospho-TYK2 (Tyr1054/1055)/TYK2 after LPS stimulation in primary HBECs: Representative Western blots of p-TYK2, TYK2, and GAPDH bands and the quantification of the ratio p-TYK2/TYK2 using one-way ANOVA with Tukey’s multiple-comparison test. Four independent experiments (*n* = 4) of primary human bronchial epithelial cell lysates obtained after pre-treatment with CBD or THC for 30 min and LPS stimulation for 8 h were analyzed, and data are expressed as means ± SD; ** *p* < 0.01, *** *p* < 0.001. (Abbreviation: HBECs: Human bronchial epithelial cells).

STAT3 is one of the most important downstream signaling molecules for many cytokines, including IL-6 and TNF-α [39,40]. Following the activation of respective cytokine receptors, tyrosine kinases, such as JAKs, phosphorylate STAT3 at Tyr-705 (*p*-STAT3), leading to its dimerization and nuclear translocation and the transcriptional activation of a variety of pro-inflammatory cytokines. We studied the ratio of expressions of total STAT3 to p-STAT3 at Tyr-705 and discovered that CBD and THC significantly decreased the upregulated ratio of t-STAT3/p-STAT3 after LPS stimulation in THP-1 macrophages and HBECs (Figure 6A,B). We also determined the phosphorylation status of STAT1 and found no significant changes in the ratio of t-STAT1/p-STAT1 in all our experimental groups (data not shown). The signaling of one of the JAKs, TYK2-mediated STAT3, has been implicated in chronic inflammatory diseases, such as cancer and Alzheimer’s disease [41,42]. A higher gene expression of *Tyk2* is also associated with severe COVID-19 in a genome-wide association study [16]. Hence, we measured the expression of the *Tyk2* gene and the phosphorylation status of TYK2 (p-TYK2) after LPS treatment. We discovered that *Tyk2* gene expression was significantly upregulated after LPS stimulation, which was efficiently downregulated by CBD and THC in THP-1 macrophages and HBECs (Figure 6C). However, surprisingly, the ratio of the expressions of p-TYK2/t-TYK2 was significantly higher in the LPS group as compared with the vehicle, and CBD or THC was able to effectively diminish this increase in p-TYK2/t-TYK2 ratio in THP-1 macrophages and HBECs (Figure 6D,E). Our immunoblotting data indicate that CBD and THC inhibit cytokine-mediated TYK2-STAT3 signaling, thus demonstrating anti-inflammatory action.

## 4. Discussion

Chronic systemic inflammation (CSI) plays a crucial role in the development of a variety of chronic human disorders. A normal acute inflammatory response is physically limited, where the insult has occurred and resolves once the insult has passed. However, the existence of several psychosomatic, biological, socioeconomic, and environmental factors has been associated not only with the avoidance of the resolution of acute inflammation but also with the promotion of a state of chronic low-grade sterile systemic inflammation categorized by stimulation of different immune mechanisms discrete from those activated during an acute immune response [43]. CSI is often triggered by sterile host-derived DAMPs, and the most common triggers include unhealthy diet; lack of sleep and disturbed circadian rhythm; physical inactivity; intestinal dysbiosis; (visceral) obesity; stress; exposure to xenobiotics, such as tobacco smoking, hazardous chemical wastes, and air pollutants; and chronic infections [43]. CSI has been associated with elevated circulating levels of cytokines and chemokines. Additionally, persistent low-grade SCI eventually results in collateral tissue and organ damage by promoting oxidative stress as one of the major mechanisms [43,44]. The evidence from meta-analyses of long-term prospective studies comprising 160,309 people showed that increased circulating levels of inflammatory biomarker C-reactive protein were linked to increased risks of coronary heart disease, ischaemic stroke, and mortality [45]. A recently published study showed that nonsurvivor COVID-19 patients had increased levels of LPS, IL-6, and TNF-α, among others [46].

NLRP3 inflammasome activation via DAMPs and oxidative stress has been implicated in increasing susceptibility to chronic inflammatory disorders, such as cardiovascular diseases [44,47]. In addition, a recent clinical trial concluded that NLRP3 inflammasome activation by antibody-mediated SARS-CoV-2 in monocytes/macrophages triggers inflammatory cell death and terminates the infection but causes systemic inflammation that contributes to COVID-19 pathogenesis [48]. Similarly, elevated baseline levels of phosphorylated STAT proteins and defective JAK-STAT signaling have been immunologically linked to chronic inflammation [49]. Inflammasome activation and IL-6-STAT3 signaling have been proven to be associated with the severity of disease in patients with COVID-19 via inducing the release of pro-inflammatory cytokines [13,50]. Although cannabinoids have documented evidence of being potent anti-inflammatory agents [51], the molecular mechanisms behind the same remain largely unknown.

The purpose of our research was to elucidate the molecular mechanisms behind the ability of major cannabinoids to inhibit the LPS-induced cytokine release in immune and non-immune cells. We aimed to fill the knowledge gap, at least partly, by providing new mechanisms of the anti-inflammatory action of CBD and THC. We selected human THP-1 macrophages as a representative immune cell model and primary HBECs as a representative non-immune cell model to study in vitro cytokine release following LPS stimulation. The reason for the use of non-immune and immune cells was to provide a clear distinction between local and systemic anti-inflammatory mechanisms, respectively. Our data revealed that pre-treatment with CBD and THC effectively decreased the expression of NLRP3 and pro-IL-1β in THP-1 macrophages and HBECs (Figure 1A,C and Figure 2A,C). The significantly lowered expression of pro-caspase-1 was observed in THP-1 macrophages (Figure 1B) but not in HBECs (data not shown). However, the result of inflammasome activation, that is, the maturation of pro-IL-1β to IL-1β, was substantially reduced by CBD and THC in THP-1 macrophages and HBECs (Figure 1D and Figure 2C). These data suggest that CBD and THC treatment alone inhibited NLRP3 inflammasome activation by reducing the expression of inflammasome-associated proteins. CBD has been previously shown to inhibit NLRP3 inflammasome activation by reducing the expression of its proteins in the livers of high-fat diet-treated mice and RAW264.7 murine macrophages [25]. However, indirect evidence shows that THC can inhibit NLRP3 inflammasome activity by reducing caspase-1 and IL-1β levels via cannabinoid 2 (CB_2_) receptor activation [5]. Hence, we showed for the first time that THC inhibits the expression of NLRP3 inflammasome proteins to block its activation in THP-1 macrophages and HBECs.

Our results demonstrated that the NF-κB-mediated transcriptional upregulation of *Nlrp3 and Il-1β* after LPS stimulation was significantly abrogated by CBD and THC in THP-1 macrophages and HBECs (Figure 3C,D). However, CBD could downregulate p-NF-κB levels in both immune and non-immune cells but not THC (Figure 3A,B). To better understand the NF-κB data in THP-1 macrophages, we individually analyzed the levels of p-NF-κB and NF-κB normalized to GAPDH due to significant changes in the expression of NF-κB after LPS stimulation (Figure 3A). However, for HBECs, NF-κB levels did not alter after LPS stimulation, so we normalized p-NF-κB levels to NF-κB for analysis (Figure 3B). These data corroborate previously published research showing that THC does not affect LPS-activated NF-κB signaling in BV-2 microglial cells [35]. Similarly, CBD has been shown to inhibit NLRP3 activation by blocking the NF-κB pathway in vivo and in vitro, ref. [25], as also shown in our data. The mechanism by which THC decreased NLRP3 activation is still unknown. Zinc-finger protein growth factor independence 1 (Gfi1) is a transcription factor that, upon activation, inhibits NLRP3 inflammasome activation in macrophages by binding to the Gli-responsive element 1 (GRE1) in the *Nlrp3* promoter [52]. Interestingly, THC has been shown to increase the gene expression of *Gfi* significantly in vivo [53]. Hence, THC might be able to inhibit NLRP3 inflammasome activation via upregulating Gfi activity, which is currently under investigation in our laboratory.

Increased circulating levels of the pro-inflammatory cytokines IL-1β, IL-6, IL-8, and TNF-α are hallmarks in the immunopathology of chronic inflammatory diseases, including COVID-19. Our IL-1β ELISA data demonstrated that high IL-1β-induced by LPS + ATP was effectively attenuated by pre-treatment with CBD and THC in cell supernatants of THP-1 macrophages and HBECs (Figure 4A). Our multiplex ELISA data showed elevated levels of IL-6, IL-8, and TNF-α after LPS stimulation, which were significantly downregulated by CBD and THC alone (Figure 4B–D). Such effects of CBD and THC or combinations of CBD and THC have been previously reported to inhibit the levels of cytokines post-LPS in vitro and in vivo [35,54,55]. Furthermore, THC treatment is beneficial in alleviating ARDS by inducing apoptosis in immune cells in a mouse model of ARDS [23]. Our data in HBECs showed that CBD and THC suppressed the elevated levels of GM-CSF post-LPS, which was congruent with previously published reports using peripheral blood mononuclear cells isolated from human blood and immune cells [56,57]. Interestingly, THC did not affect the increased IL-10 levels induced by LPS in THP-1 macrophages, whereas CBD significantly reduced its levels post-LPS (Figure 4E). We believe that because of the significant cytokine storm induced by LPS (higher levels of IL-6, IL-8, and TNF-α) in THP-1 macrophages, the levels of the anti-inflammatory cytokine IL-10 were increased as a compensation mechanism, indicating an imbalance between pro- and anti-inflammatory cytokines. THC, in this case, proved to be a more potent anti-inflammatory cannabinoid than CBD (Figure 4E). Such an effect of THC-induced increase in IL-10 levels has been previously reported in monocytic myeloid-derived cells of endotoxemic mice via the activation of CB_1_ receptors, leading to lung protection [58]. Oxidative stress-induced tissue and organ damage occurring simultaneously with cytokine surge is highly characteristic in the pathophysiology of chronic lung disorders and COVID-19 [59,60]. Our cellular ROS generation assay revealed that CBD and THC substantially attenuated oxidative stress induced by LPS + ATP in THP-1 macrophages and HBECs without being cytotoxic to these cells (Figure 5A,B). Our results are consistent with published reports showing that CBD and THC possess considerable antioxidant properties [61,62].

As mentioned earlier, the cytokine-activated JAK-STAT pathway, especially the IL-6-STAT3 pathway, has been linked to the induction of pro-inflammatory cytokines and the pathophysiology of COVID-19 [12,13]. Our immunoblotting data revealed that CBD and THC effectively diminished the increased p-STAT3/t-STAT3 ratio after LPS stimulation in THP-1 macrophages and HBECs (Figure 6A,B). Our qRT-PCR results demonstrated that CBD and THC significantly reduced the elevated expression of *Tyk2*, a JAK implicated in the severity of COVID-19 [16], post-LPS treatment in THP-1 macrophages and HBECs (Figure 6C). However, no changes were observed in the protein expression of TYK2 in primary HBECs, and on the contrary, the protein expression of TYK2 in THP-1 macrophages was significantly reduced after LPS stimulation (Figure 6A). This finding led us to analyze phosphorylation of TYK2 (p-TYK2), and we found that the ratio of p-TYK2/TYK2 was significantly higher after LPS treatment in both types of cells, which was significantly reduced by CBD and THC (Figure 6D,E). Although we have not investigated this mechanism, TNF-α has been shown to activate JAK1 and TYK2 in human B cells to further stimulate JAK1-STAT3 signaling via TNF-receptor-1 [39]. Additionally, IL-6 and TNF-α have both been shown to activate STAT3 and NF-κB in human T cells to promote inflammatory tumor growth [63]. CBD and THC have been proven to be effective in mice models of lung inflammation and ARDS [23,64]. Hence, our data, along with other published literature, strongly suggest that CBD and THC may be protective in lung inflammation by inhibiting IL-6/TYK2/STAT3 pathways in HBECs; however, the contribution from other JAKs in this regard remains to be investigated.

## 5. Limitations and Future Directions

Our study has several limitations that must be considered before interpreting the results. Human THP-1 macrophages behave similarly to freshly isolated primary human peripheral blood monocyte-derived macrophages (MDMs); however, changes in gene expression after LPS stimulation markedly differ between them [65]. THP-1 cells are still very widely used as an in vitro cell model to study inflammatory signaling. Nevertheless, the experiments in this research work should be validated in MDMs or freshly isolated murine monocytes/macrophages to reach definitive conclusions. Next, our focus in this work was to decipher the role of cannabinoids in the regulation of anti-inflammatory pathways. As shown in primary human skin keratinocytes and primary human dermal fibroblasts, LPS stimulation increases the mRNA expression of CB_1_ and CB_2_ [66]. The expression of cannabinoid receptors is very likely to have been affected by LPS in our studies. Such changes may have noticeable effects, especially with respect to THC pharmacology. However, we were interested in studying cannabinoid receptor-independent mechanisms; hence, CB_1_/CB_2_-specific ligands were not employed. Third, we pretreated our cells with CBD and THC, as published elsewhere [21]; however, for future in vivo experiments, the treatment of chronic inflammation can be mimicked by treating animals with cannabinoids after the induction of inflammation. Lastly, in the future, the contribution of different JAKs in inhibiting STAT3 signaling and transcription factors modulated by THC can be identified by performing RNA sequencing. The role of cannabinoids on NLRP3 inflammasome assembly can be elucidated by studying protein–protein interactions between NLRP3 inflammasome-associated proteins via co-immunoprecipitation, surface plasmon resonance, or mass spectrometry.

## 6. Conclusions and Clinical Implications

**Figure 7:**
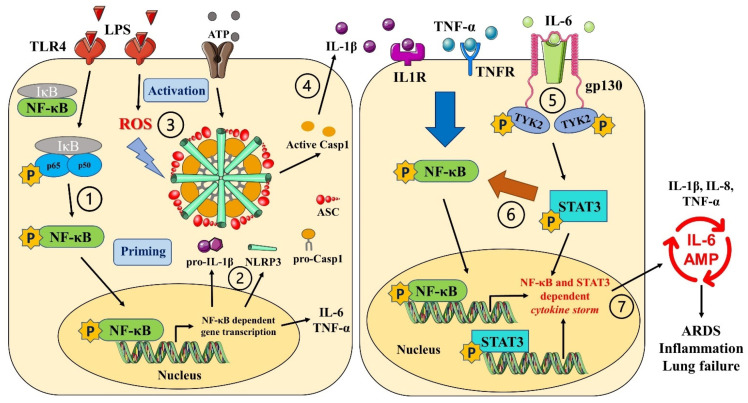
Summary of anti-inflammatory pathways activated by CBD and THC to curb LPS-induced cytokine storm: ①. LPS activates TLR4 receptors which in turn phosphorylates NF-κB. Phosphorylated NF-κB moieties translocate to the nucleus, thereby upregulating the expression of variety of pro-inflammatory genes. ②. This TLR4-NFκB-mediated “priming” (increased expression of NLRP3, pro-IL-1β, and pro-caspase-1) is the first step in the activation of NLRP3 inflammasome. ③. LPS also induces oxidative stress causing higher levels of ROS which in turn act as second “activation” signal leading to NLRP3 inflammasome assembly and activation. ④. The NLRP3 inflammasome activation causes the release of mature IL-1β leading to cell death via pyroptosis. ⑤. Along with inflammasome activation, NFκB activation also increases the levels of variety of other pro-inflammatory cytokines including IL-6. IL-6 binds to its receptors coupled to transmembrane protein, gp130, which in turn, in part, phosphorylates TYK2. ⑥. Phosphorylated TYK2, in turn, phosphorylates STAT3 leading to the translocation of the latter to the nucleus. Phosphorylated STAT3 furthermore potentiates NFκB activation. ⑦. Nuclear translocation of STAT3 and NFκB activate gene expression of various pro-inflammatory cytokines leading to cytokine release syndrome or cytokine storm. This cytokine storm activates vicious cycle of IL-6 amplification (IL-6 AMP) which is implicated in chronic inflammation, tissue injury, and organ damage. Our in vitro data revealed that CBD and THC block all the steps numbered in the figure above in both THP-1 macrophages (immune cells) and primary human bronchial epithelial cells (non-immune cells).

Chronic inflammatory disorders are accountable for more than 50% of deaths worldwide. The understanding and targeting of crucial inflammatory signaling pathways could substantially support current therapy to improve patients’ quality of life. Our research demonstrated that cannabinoids, particularly CBD and THC, inhibit NLRP3 inflammasome signaling to attenuate the elevated levels of IL-1β released by LPS + ATP in THP-1 macrophages and primary HBECs. The possible mechanisms behind their action are via inhibiting NF-κB priming and reducing cellular ROS generation (Figure 7). Moreover, LPS-induced release of the pro-inflammatory cytokines IL-6, IL-8, and TNF-α was also significantly weakened by CBD and THC, in part by inhibiting IL-6/TYK2/STAT3 pathway in both THP-1 macrophages and primary HBECs (Figure 7). Our data strongly suggest that CBD and THC can be developed as key therapeutic molecules to substantially diminish inflammasome activation and IL-6-STAT3 signaling both locally at the level of lungs and systemically in immune cells to support the current treatment of chronic inflammatory disorders. These data must also be explored to consider CBD, THC, and/or their combination as a strategy to curb cytokine storm during ARDS in COVID-19.

## Figures and Tables

**Table 1:** Primary antibodies with manufacturers and dilutions.

Primary Antibody	Manufacturer (Cat#)	Dilution
NLRP3	CST (15101S)	1:750
Caspase-1	CST (4199S)	1:500
IL-1β	SCBT (sc-32294)	1:500
NFκB p65	SCBT (sc-8008)	1:750
phospho-NFκB p65 (Ser-536)	CST (13346S)	1:750
STAT-3	SCBT (sc-8019)	1:750
phospho-STAT3 (Tyr-705)	SCBT (sc-8059)	1:750
STAT-1	Abcam (ab109320)	1:2000
phospho-STAT1 (Ser-727)	Abcam (ab109461)	1:2000
TYK2	CST (14193S)	1:1000
phospho-TYK2 (Tyr-1054/1055)	CST (9321S)	1:500
GAPDH	SCBT (sc-32233)	1:1000
β-actin	SCBT (sc-47778)	1:1000

**Table 2:** List of primers used for qRT-PCR.

Gene Name	Forward Primer	Reverse Primer
*Nlrp3*	GAAGAGGAGTGGATGGGTTTAC	TCTGCTTCTCACGTACTTTCTG
*Il-1β*	CCTTAGGGTAGTGCTAAGAGGA	AAGTGAGTAGGAGAGGTGAGAG
*Tyk2*	CAAATGTCCCTGTGAGGTCTATC	GGACTGTCTTCAGAATGGGTATG
*Gapdh*	CAGGAGGCATTGCTGATGAT	GAAGGCTGGGGCTCATTT

## Data Availability

The raw data used and/or analyzed during the current study are available from the corresponding author(s) upon reasonable request.
